# Clinical profile and early therapeutic response to cabergoline of patients with hyperprolactinemia in a Cameroonian population

**DOI:** 10.11604/pamj.2020.35.2.12883

**Published:** 2020-01-02

**Authors:** Martine Claude Etoa Etoga, Eugène Sobngwi, Pelagie Ngoune, Emmanuella Doh, Francine Mendane Mekobe, Noel Mbango-Ekouta, Mesmin Dehayem, Pascal Foumane, Jean Claude Mbanya

**Affiliations:** 1 Department of Clinical Sciences, Faculty of Medicine and Pharmaceutical Sciences, University of Douala, Douala, Cameroon; 2 National Obesity Centre, Diabetes and Metabolic Diseases Unit, Yaounde Central Hospital, Yaounde, Cameroon; 3 Department of Internal Medicine and Specialties, Faculty of Medicine and Biomedical Sciences, University of Yaounde 1, Yaounde, Cameroon; 4 Internal Medicine Unit, Centre Hospitalier d´Essos, Yaounde, Cameroon; 5 Department of Obstetrics and Gynecology, Faculty of Medicine and Biomedical Sciences, University of Yaounde 1, Yaounde, Cameroon

**Keywords:** Hyperprolactinemia, cabergoline, early therapeutic response

## Abstract

Hyperprolactinemia is responsible for 20 to 25% of consultations of secondary amenorrhea and 17% for female infertility. Dopamine agonists are the gold standard treatment of hyperprolactinemia. Although they are associated with various adverse effects, cabergoline is generally preferred due to better compliance, limited side effects and good therapeutic response. However, bromocriptine is widely and satisfactorily used in a context of limited availability of cabergoline. We sought to describe clinical manifestations of hyperprolactinemia and response to cabergoline in a sub Saharan Africa (SSA) setting. We describe the profile of all patients with a diagnosis of hyperprolactinaemia from 1^st^ July 2012 to 15^th^ May 2014 at the Endocrinology Department of Yaoundé Central Hospital. Patients with physiological hyperprolactinemia were not considered. All patients were routinely started on cabergoline at 0.5mg/week or at 1mg/week in case of macroprolactinoma or desire to become pregnant. The duration of follow up was 8-16 months. After three months of treatment, 8 of 10 patients with amenorrhea had menses and serum prolactin levels decreased significantly at month 2-3 (p = 0.025). In conclusion, our study suggests that cabergoline yields an excellent therapeutic response in a short period of time and may thus be cost saving in sub Saharan context despite its unit price.

## Introduction

Hyperprolactinemia is one of the most frequent disorders of the pituitary gland. It is responsible for 20 to 25% of consultations of secondary amenorrhea [1] and 17% for female infertility [2], and can be caused by physiological, pharmacological or pathologic factors. Clinical presentations of hyperprolactinemia vary with study populations and depends on the etiology hence its profiling in different populations is necessary [3]. There is paucity of data concerning sub Saharan Africa (SSA). The clinical picture in this context may reflect both difficult access to care and possible ethnic/geographic specificities. The impact on the growing public health problem of infertility in Africa is unknown. Dopamine agonists are the gold standard treatment of hyperprolactinemia, although associated with various adverse effects. Cabergoline is generally preferred due to better compliance, limited side effects and good therapeutic response [4, 5]. Bromocriptine widely and satisfactorily used in a context of limited availability of cabergoline [6]. Whether the more expensive cabergoline would be effective and potentially cost saving has not been reported. We sought to describe clinical manifestations of hyperprolactinemia and response to cabergoline in a SSA setting, as improving on the diagnosis and management for hyperprolactinemia is likely to improve infertility care in this context.

## Methods

We conducted a cross-sectional study over a period of 2 years. We describe the profile of all patients with a diagnosis of hyperprolactinaemia at the Endocrinology Department of Yaoundé Central Hospital, Yaoundé. Patients with physiological hyperprolactinemia including pregnant and lactating women were not considered. Baseline work up included clinical data collection using a pre-designed questionnaire, the assay of prolactinemia by electro-chemiluminescence, ultra-sensitive thyroid-stimulating hormone (TSH), blood urea nitrogen, serum creatinine and liver function panel. Hypothalamo-pituitary MRI or CT scan was performed in all participants. All patients were routinely started on cabergoline at 0.5mg/week or at 1mg/week in case of macroprolactinoma or desire to become pregnant. Patients were monitored on a monthly basis until 5 months after initiation of the treatment then every 3-months, to assess compliance and drug tolerance using a literature-derived checklist of possible adverse symptoms or physical signs. All participants gave their informed written consent.

## Results

Participants included 24 females and 1 male aged 24 to 58 years with a mean of 37.1±8.9 years. Clinical features at presentation included mainly infertility (20/25), galactorrhea (18/25), menstrual disturbances (16/24), visual defects (4/25), and erectile dysfunction in the only male patient. The most probable causes of hyperprolactinaemia were microadenoma in 13/25, macroprolactinoma in 4/25, non-secreting macroadenoma in 4/25. No cause was identified in 4/25 cases suggesting missed microadenoma or idiopathic hyperprolactinaemia. All patients were treated with cabergoline and one non-secreting macroadenoma with visual signs required additional surgical treatment. The duration of follow up was 8-16 months. After three months of treatment, 8 of 10 patients with amenorrhea had menses, 12/18 had no more galactorrhea. Prolactin levels normalized in 16/25. Fertility was restored with confirmed pregnancy in 7/17 by the 16th month of follow up. Serum prolactin levels decreased significantly at month 2-3 (p = 0.025). Table 1 shows temporal changes in clinical symptoms and signs under treatment. Concerning adverse effects, 5/25 patients had transient dizziness, and 4 complained of headache that subsided spontaneously after two weeks of treatment without dose change. The other reported symptoms included digestive tract disorder (4/25), asthenia (3/25), oedema (1/25), and alopecia (1/25).

**Table 1:** Temporal changes in clinical characteristics under cabergoline treatment

Clinical signs	At diagnosis	Durations of treatment (in months)						
		01	02	03	04	05	06 to 12	>12
Amenorrhoea	10	7/10	4/10	2/10				
Spaniomenorrhoea	06	4/6	2/6	2/6	1/6			
Oligomenorrhoea	05	5/5	2/5	1/5	0/5			
Galacthorrhoea	18	10/18	6/18	5/18	4/18	4/18	2/18	2/18
Breast engorgement	13	8/13	4/13	0/13				
Vaginal dryness	08	6/8	4/8	3/8				1/8
Dyspareunia	07	5/7	3/7	2/7				0/7
Headaches	12	11/12	9/12	3/12	2/12	1/12		
Bitemporal hemianopia	03	3/3	2/3	1/3				
Decrease of visual acuity	02	2/2	1/2	1/2	0/2			
Decrease of libido	07	2/7	2/7	1/7	1/7	1/7	1/7	0/7
Erectile dysfunction	01	1/1	1/1	1/1	1/1	1/1	1/1	1/1
Vomitting	01	0/1	0/1					
Infertility	20	20/20	20/20	19/20	19/20	18/20	16/20	13/20
Pregnancy desire	17	17/17	17/17	16/17	16/17	15/17	13/17	10/17

## Discussion

In this case series in a SSA population with confirmed pathological hyperprolactinaemia and most of patients referred by gynaecologists, there was a clear female predominance with infertility and galactorrhea being the most frequent presenting complains. According to WHO, one in every four couple in developing countries have been found to be affected by infertility [7]. A study carried out in 2012 stated that the prevalence of infertility was highest in South Asia, Sub-Saharan Africa, North Africa/Middle East, Central/Eastern Europe and Central Asia [8]. In SSA, infertility is becoming a major public health problem with strong cultural and psychosocial implications. Infertility is multifactorial with the most frequent contributor to female infertility being tubopelvic diseases [5]. The role played by hyperprolactinemia remains unknown and there is paucity of data in sub Saharan populations. Dopamine agonists are the gold standard approach for the treatment of hyperprolactinemia with good therapeutic response in general. Cabergoline and Bromocriptine are the two major ones [9]. In SSA context; most studies reported results only with Bromocriptine for availability and access reasons. In studies comparing cabergoline and bromocriptine, side effects were less frequent and severe with cabergoline [5]. In our cohort we used solely cabergoline with one case requiring an additional surgical treatment. This treatment was associated with an excellent therapeutic response as from 2 to 3 months of use despite a few minor and transient side effects most of which subsided in about 12 weeks. The promising results on fertility within a very short term of 41% compared to 28% within an average of 26 months in a study in Thailand that used mostly bromocriptine [10], suggest a more prominent role of hyperprolactinaemia in infertility in our cohort, or a better response to cabergoline or both.

## Conclusion

Our study suggests that serum prolactin levels should be measured in all patients consulting for infertility or for menstrual cycle disorders, and that cabergoline yields an excellent therapeutic response in a short period of time and may thus be cost saving in sub Saharan context despite its unit price.

### What is known about this topic

- Hyperprolactinemia is a main cause of infertility even in men and women;
- Prolactinomas are the main causes of hyperprolactinemia;
- There is a good therapeutic response to cabergoline.

### What this study adds

- The study highlights the early therapeutic response and in a short time of cabergoline;
- Microprolactinomas is most frequent in our context;
- Hyperprolactinemia is also a frequent cause of infertility in our context.

## Competing interests

The authors declare no competing interests.
